# Celastrol Inhibited Human Esophageal Cancer by Activating DR5-Dependent Extrinsic and Noxa/Bim-Dependent Intrinsic Apoptosis

**DOI:** 10.3389/fphar.2022.873166

**Published:** 2022-06-08

**Authors:** Xihui Chen, Shiwen Wang, Li Zhang, Shuying Yuan, Tong Xu, Feng Zhu, Yanmei Zhang, Lijun Jia

**Affiliations:** ^1^ Cancer Institute, Longhua Hospital, Shanghai University of Traditional Chinese Medicine, Shanghai, China; ^2^ Department of Laboratory Medicine, Huadong Hospital Affiliated to Fudan University, Shanghai, China

**Keywords:** celastrol, esophageal squamous cell carcinoma (ESCC), tumor growth, extrinsic apoptosis, intrinsic apoptosis

## Abstract

Esophageal squamous cell carcinoma (ESCC) is one of the deadliest digestive system cancers worldwide lacking effective therapeutic strategies. Recently, it has been found that the natural product celastrol plays an anti-cancer role in several human cancers by inducing cell cycle arrest and apoptosis. However, it remains elusive whether and how celastrol suppresses tumor growth of ESCC. In the present study, for the first time, we demonstrated that celastrol triggered both extrinsic and intrinsic apoptosis pathways to diminish the tumor growth of ESCC *in vivo* and *in vitro*. Mechanistic studies revealed that celastrol coordinatively induced DR5-dependent extrinsic apoptosis and Noxa-dependent intrinsic apoptosis through transcriptional activation of ATF4 in ESCC cells. Furthermore, we found that the FoxO3a-Bim pathway was involved in the intrinsic apoptosis of ESCC cells induced by celastrol. Our study elucidated the tumor-suppressive efficacy of celastrol on ESCC and revealed a previously unknown mechanism underlying celastrol-induced apoptosis, highlighting celastrol as a promising apoptosis-inducing therapeutic strategy for ESCC.

## Introduction

Esophageal cancer is one of the most aggressive digestive system cancers globally (Bray et al., 2018). Esophageal squamous cell carcinoma (ESCC) is the main histologic subtype among all types of esophageal tumors, which is more prevalent in East Asia (Huang and Fu, 2019). At present, surgery combined with neoadjuvant chemotherapy and radiotherapy is the first-line treatment for ESCC (Watanabe et al., 2019). Major limitations of the treatment of ESCC include high toxicity and acquired therapeutic resistance to chemotherapy and radiotherapy, as well as the high recurrence rate of surgery (Leng et al., 2020). In recent years, although some clinical advances have been made in the development of diagnosis and therapeutic techniques, the overall 5-year survival rate for ESCC patients is still very poor (Han et al., 2022). Consequently, it is pressingly needed to facilitate the development of effective strategies for ESCC therapy.

Recently, natural products have been increasingly used in the treatment and prevention of human cancers due to their significant efficacy and few side effects (Deng et al., 2020; Ma et al., 2021; Yang et al., 2021). A variety of natural products were confirmed to exert anti-ESCC activity by inducing apoptosis, regulating autophagy, arresting the cell cycle, and inhibiting metastasis (Ying et al., 2018). For example, the natural product berberine was found to inhibit the proliferation of ESCC cells by promoting cell cycle arrest at the G2/M phase (Jiang et al., 2017). Furthermore, echinatin, a compound isolated from the Chinese herb Glycyrrhiza uralensis Fisch, suppressed the growth and invasion of ESCC cells by inducing AKT/mTOR-dependent autophagy and apoptosis (Hong et al., 2020). In addition, the herbal ingredient artesunate inhibited the migration of ESCC cells by interfering with DNA synthesis and destroying the cytoskeleton (Shi et al., 2015). Therefore, natural products exhibited substantial anti-ESCC efficacy, which was expected to provide a new direction for the clinical treatment of ESCC.

**FIGURE 1:**
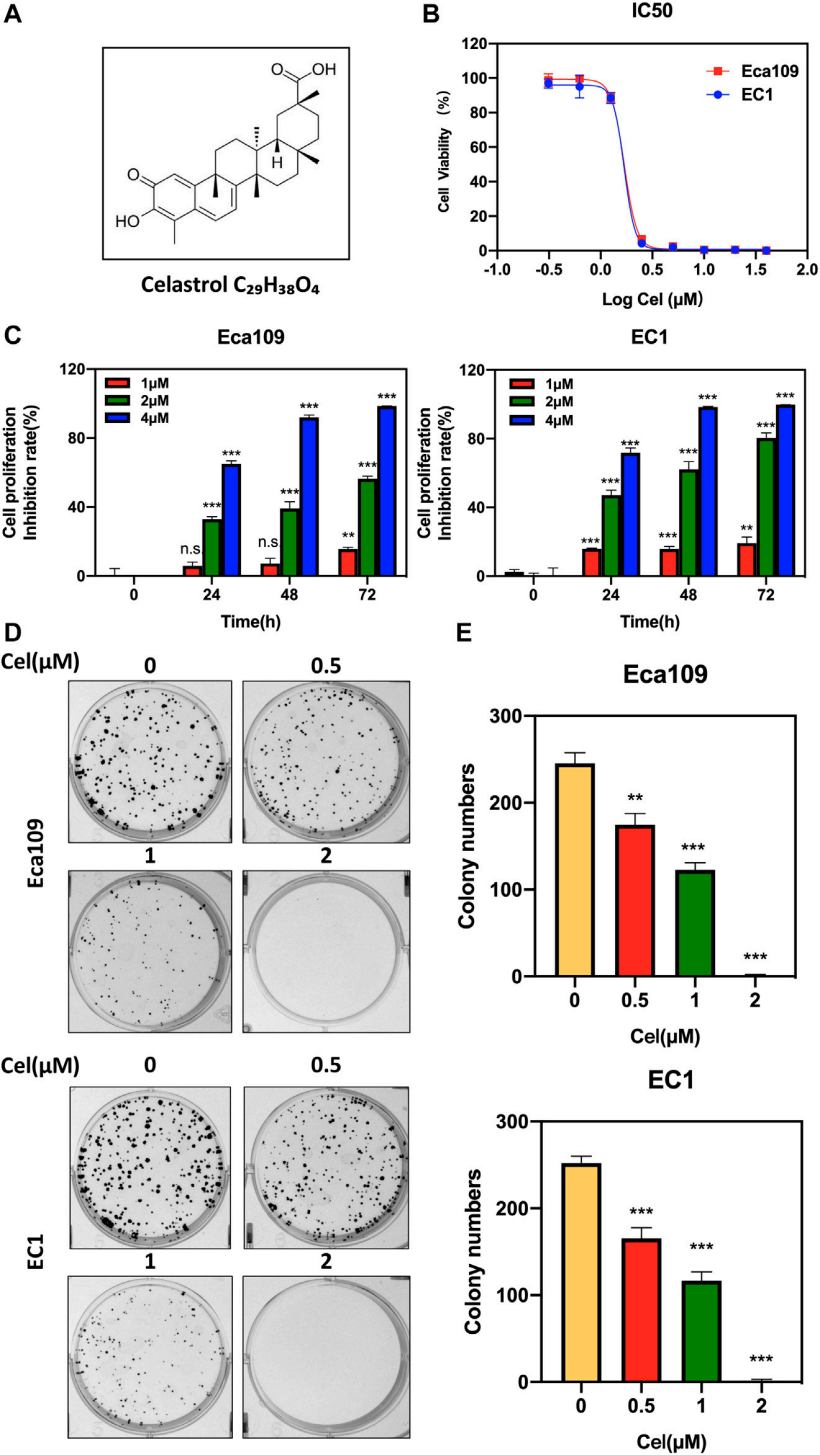
Celastrol inhibited the viability of ESCC cells. **(A)** Chemical structure of celastrol. **(B)** Human ESCC cells Eca109 and EC1 were treated with indicated concentrations of celastrol for 72 h, and cell viability was determined by ATPlite assay. Representative inhibitory curves for each cell line were shown. **(C)** ATPlite assay was used to determine the cell growth of Eca109 and EC1 cells at the indicated concentration of celastrol or DMSO for 0, 24, 48, and 72 h **(D,E)** Representative images of three independent experiments were shown for the inhibition of colony formation by 0.5, 1, and 2 μM celastrol or DMSO for 10 days. Graphs of the relative number of colonies were performed. ^∗^^∗^denotes *p* < 0.01, ^∗^^∗^^∗^denotes *p* < 0.001, n.s. denotes not significant.

The natural product celastrol is a kind of pentacyclic triterpene extracted from the herbaceous plant Tripterygium wilfordii Hook F (TWHF) (Figure 1A), which possesses anti-inflammatory, anti-rheumatic, and some other pharmacological activities (Yang et al., 2017; Zhang et al., 2017; Tang et al., 2018; Saito et al., 2019; Wong et al., 2019; Ye et al., 2020). Recent studies have shown that celastrol exerts potential anti-cancer activity in various human cancers, including breast cancer, gastric cancer, lung cancer, and colorectal cancer (Dharambir et al., 2018). It was reported that celastrol inhibited breast cancer cell's metastasis by intervening M2-like polarization by inhibiting STAT6 (Yang et al., 2018). Moreover, celastrol was found to suppress nitric oxide (NOS) synthases and the angiogenesis pathway, thereby inhibiting the growth and migration of colorectal cancer cells (Gao et al., 2019). However, the tumor-suppressive efficacy of celastrol on ESCC and the underlying mechanisms remain largely undefined.

Inducing apoptosis is an effective way to prevent and treat human cancers (Carneiro and El-Deiry, 2020; Shahar and Larisch, 2020). Extrinsic (death receptor-mediated) apoptosis and intrinsic (mitochondrial) apoptosis represent the most important cytotoxic pathways activated by anti-cancer agents (Kale et al., 2017). In extrinsic apoptosis, death receptors, such as Fas and TRAIL, interact with their specific ligands to trigger apoptosis cascades by recruiting and activating the main downstream factor caspase 8 (Daolin et al., 2019). Intrinsic apoptosis is activated by the release of cytochrome c in mitochondria and the cleavage of caspase 9, and is regulated by the balance between pro-survival and pro-apoptotic Bcl-2 protein family members (Diepstraten et al., 2022). So far, the underlying mechanisms of celastrol triggering ESCC apoptosis are still unclarified. Here, for the first time, we validated the anti-tumor activity of celastrol in ESCC both *in vivo* and *in vitro*. Mechanistically, we revealed that celastrol suppressed the tumor growth of ESCC by activating DR5-dependent extrinsic and Noxa/Bim-dependent intrinsic apoptosis. Our findings not only elucidated the tumor-suppressive efficacy of ESCC and its underlying mechanism but also provided preliminary evidence for the clinical treatment of ESCC by celastrol.

## Materials and Methods

### Cell Culture

Human ESCC cell lines Eca109 and EC1 were purchased from the Type Culture Collection of the Chinese Academy of Sciences (Shanghai, China), and cultured in media at 37°C with 5% CO_2_. All media consisted of Dulbecco’s Modified Eagle’s Medium (DMEM, BasalMedia, Shanghai, China), 10% fetal bovine serum (FBS, Biochrom AG, Berlin, Germany), and 1% penicillin-streptomycin solution (BasalMedia, Shanghai, China).

### Reagents

Celastrol was acquired from MCE (MedChem Express, Shanghai, China), and the purity of the compounds was ≥99.65%. Celastrol was dissolved in dimethyl sulfoxide (DMSO), and DMSO was used as the vehicle control. For *in vivo* study, celastrol was dissolved first in DMSO and then in 10% 2-hydroxypropyl-β-cyclodextrin (Sangon Biotech, Shanghai, China).

### Antibody

Primary antibodies to the following proteins were used: cleaved PARP (c-PARP), cleaved caspase 8 (c-caspase 8), cleaved caspase 9 (c-caspase 9), ATF4, CHOP, Noxa, DR3, Bax, Bad, Bid, Bim, p53, p21, p-histone 3 (p-H3), p-H2AX, p-cdc2 (Cell Signaling Technology, Danvers, MA, United States); DR5 and FoxO3a (Abcam, Cambridge, MA, United States); TNFR1, TNFR2 and β-actin (HuaBio, China).

### Cell Viability Assay

For cell viability assay, cells were seeded in black 96-well plates with 2×10^3^ cells per well in triplicate and allowed to attach overnight. Cells were treated with DMSO, celastrol, Z-VAD-FMK (MedChem Express, Shanghai, China), or both celastrol and Z-VAD-FMK at the indicated concentrations for the indicated time. According to the manufacturer’s protocol, the cell proliferation was measured by ATPlite luminescence assay (PerkinElmer, Norwalk, CT, United States) at the end of the incubation. The IC_50_ values were measured by the Logit method.

### Clonogenic Survival Assay

For clonogenic survival assay, cells were seeded in six-well plates with 400 cells per well in triplicate and allowed to attach overnight. Cells were treated with DMSO or celastrol at the indicated concentrations and cultured for 10 days. At the end of incubation, cells were stained with crystal violet. Colonies with more than 50 cells each were counted and photographed with a gel imager (GelDoc XR System, Bio-rad, United States).

### Apoptosis Assay

For apoptosis assay, cells were seeded at a density of 2.5 × 10^5^ cells per well in six-well plates and allowed to attach overnight. Cells were exposed to DMSO or celastrol for 24 h and stained with AnnexinV-FITC and PI Apoptosis Kit according to the manufacturer’s protocol (Share Biotechnology, Shanghai, China). Data were collected and analyzed using a flow cytometer (Beckman Coulter CytoFLEX, CA, United States).

### Cell Cycle Analysis

For cell cycle analysis, cells were seeded at a density of 2.5 × 10^5^ cells per well in six-well plates and allowed to attach overnight. Cells were exposed to DMSO or celastrol for 24 h. And then cells were harvested and fixed in 70% ice-cold ethanol at −20°C overnight. The samples were incubated in propidium iodide (PI, 36 mg/ml; Sigma, St. Louis, MO, United States) for 15 min at 37°C. The cells were detected by flow cytometer (Beckman Coulter CytoFLEX, State of California, United States). Data of cell cycle were analyzed with FlowJo 8 software.

### Western Blot Analysis

Total protein from cultured cells and tumor tissues was collected by using RIPA (Radio Immunoprecipitation Assay) lysis buffer, and protein concentration was quantified using a BCA protein assay kit (Vazyme Biotech, Nanjing, China). 20–40 mg protein was resolved by 7.5–15% SDS-PAGE, followed by electro-transferred to an Immobilon-PVDF Membrane (Merck Millipore Ltd, Tullagreen, Ireland). The membrane was then blocked with 5% skim milk for 1 h at room temperature. After being washed three times with TBST, PVDF membranes were incubated with primary antibodies at 4°C overnight. After washing, corresponding second antibodies were incubated with membranes for 1 h at room temperature, and the membranes were photographed by Tanon 5200 visualizer (Tanon, Shanghai, China).

### Real-Time Polymerase Chain Reaction Analysis

According to the manufacturer’s instructions, total RNA was isolated by using the Ultrapure RNA kit (ComWin Biotech, Beijing, China). Total RNA was purified and reversed to cDNA by using the PrimerScript reverse transcription reagent kit (Vazyme Biotech, Nanjing, China). The cDNA was quantified with RT-PCR by using the Power SYBR Green PCR MasterMix (Vazyme Biotech, Nanjing, China) on the ABI 7500 thermocycler (Applied Biosystems, Foster City, CA, United States). The mRNA data of each sample were normalized to β-actin. ATF4, CHOP, DR5, Noxa, Bim, and FoxO3a are encoded by *ATF4*, *CHOP* (*DDIT3*), *DR5* (*TNFRSF10B*), *NOXA* (*PMAIP1*), *BIM* (*BCL2L11*), and *FOXO3* genes, respectively. The sequences of the primers were as follows: human *β-actin*: forward 5′-CGT​GCG​TGA​CAT​TAA​GGA​GAA​G-3′; and reverse 5′-AAG​GAA​GGC​TGG​AAG​AGT​GC-3′; human *ATF4*: forward 5′-ATG​ACC​GAA​ATG​AGC​TTC​CTG-3′, and reverse 5′-GCT​GGA​GAA​CCC​ATG​AGG​T-3′; human *CHOP*: forward 5′-AGC​CAA​AAT​CAG​AGC​TGG​AA-3′, and reverse 5′-TGG​ATC​AGT​CTG​GAA​AAG​CA-3′; human *DR5*: forward 5′-CCA​GCA​AAT​GAA​GGT​GAT​CC-3′, and reverse 5′-GCA​CCA​AGT​CTG​CAA​AGT​CA-3′; human *NOXA*: forward 5′-ACC​AAG​CCG​GAT​TTG​CGA​TT-3′, and reverse 5′-ACT​TGC​ACT​TGT​TCC​TCG​TGG-3′; human *BIM*: forward 5′-TAA​GTT​CTG​AGT​GTG​ACC​GAG​A-3′, and reverse 5′-GCT​CTG​TCT​GTA​GGG​AGG​TAG​G-3′; human *FOXO3*: forward 5′-CAG​CCA​GTC​TAT​GCA​AAC​CC-3′, and reverse 5′-ATC​CAA​CCC​ATC​AGC​ATC​CA-3′.

### siRNA Silencing

The cells were transfected with siRNA oligonucleotides by using Lipofectamine 2000 (Invitrogen, United States). Opti-MEM (Invitrogen, United States) was used to incubate with siRNA and Lipofectamine 2000 according to the manufacturer’s instructions. All siRNAs were synthesized by GenePharma (Shanghai, China). The sequences of siRNA were as follows: si*Control*: 5′-UUC​UCC​GAA​CGU​GUC​ACG​UTT-3′; si*ATF4*-1: 5′-CCC​UUC​AGA​UAA​UGA​UAG​UTT-3′; si*ATF4*-2: 5′-CCT​CAC​TGG​CGA​GTG​TAA​A-3′; si*DR5*-1: 5′-AAG​ACC​CUU​GUG​CUC​GUU​GUC-3′; si*DR5*-2: 5′-CAG​CCG​UAG​UCU​UGA​UUG​UTT-3′; si*NOXA*-1: 5′-GGU​GCA​CGU​UUC​AUC​AAU​UUG​TT-3′; si*NOXA*-2: 5′-CCG​GCA​GAA​ACU​UCU​GAA​UTT-3′; si*BIM*-1: 5′-UCU​UAC​GAC​UGU​UAC​GUU​AUU-3′; si*BIM*-2: 5′-CAA​CCA​CUA​UCU​CAG​UGC​A-3′; si*FOXO3*-1: 5′-GGA​ACG​UGA​UGC​UUC​GCA​ATT-3′; si*FOXO3*-2: 5′-AGG​GAA​GUU​UGG​UCA​AUC​ATT-3′.

### *In Vivo* Xenograft Model

Animal experiments were performed in accordance with the National Guidelines for Experimental Animal Welfare, with approval from the Institutional Animal Care and Use Committee of Longhua Hospital, Shanghai University of Traditional Chinese Medicine. Five-week-old, BALB/c nude female mice were purchased from Lingchang Biological Technology Co., Ltd. (Shanghai, China). Mice were kept and bred at a constant room temperature with a 12:12 h light/dark cycle and fed a standard rodent diet and water. 2 × 10^7^ Eca109 cells were subcutaneously injected into the bilateral flank of each mouse. Then, mice were randomly divided into three experimental groups (*n* = 5): control, 4 mg/kg celastrol treatment group, and 8 mg/kg celastrol treatment group. Mice were treated with either β-cyclodextrin crystalline (vehicle control) or celastrol (4 or 8 mg/kg) via intraperitoneal injection every other day. Tumor volumes were determined by measuring length (l) and width (w) and calculating volume (V = 0.5 × l × w^2^) every other day. Mice were sacrificed, and tumor tissues were weighed and photographed.

### Statistical Analysis

The statistical significance of differences between groups was assessed using GraphPad Prism 8 software (San Diego, CA, United States). All data from three independent experiments were expressed as mean ± SEM. The student’s t-test was used for the comparison of parameters between groups. *p*-value of *p* < 0.05 was significant, n.s = not significant. For all tests, three levels of significance (*p < 0.05, **p < 0.01, ***p < 0.001) were used.

## Results

### Celastrol Inhibited the Viability of Esophageal Squamous Cell Carcinoma Cells

To evaluate the effect of celastrol on the proliferation of ESCC cells, we first examined the IC_50_ values of celastrol on two ESCC cell lines Eca109 and EC1. The IC_50_ values of celastrol on Eca109 and EC1 were 1.688 and 1.684 μM, respectively (Figure 1B). Furthermore, we found a time and dose-dependent growth inhibitory efficacy in two ESCC cell lines upon celastrol treatment (Figure 1C). In addition, our results showed that celastrol significantly inhibited the colony formation of both two ESCC cell lines in a dose-dependent manner (Figures 1D,E). Therefore, these findings demonstrated celastrol obviously inhibited the viability of ESCC cells.

### Celastrol Induced Apoptosis in Esophageal Squamous Cell Carcinoma Cells

**FIGURE 2:**
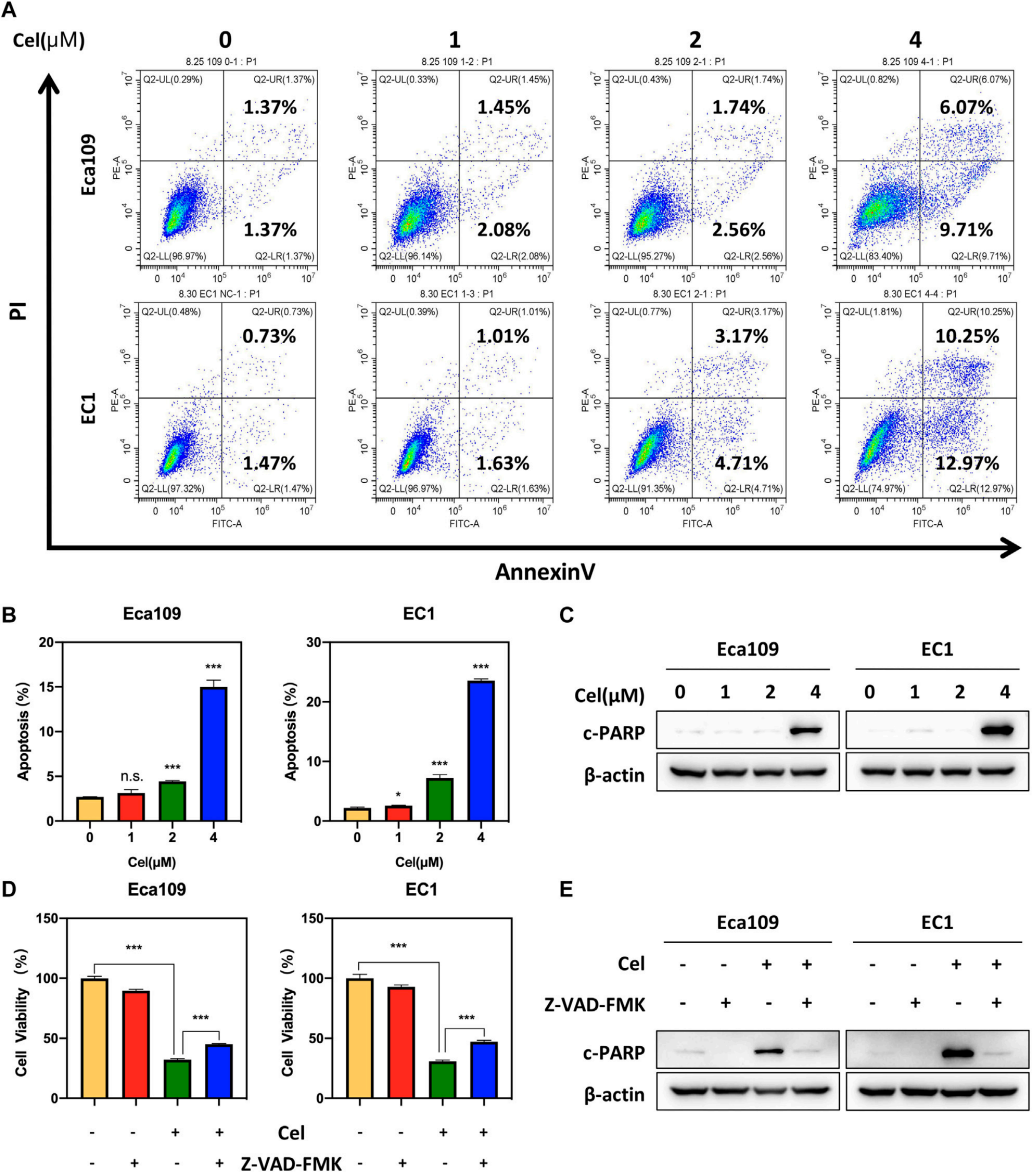
Celastrol induced apoptosis in ESCC cells. **(A,B)** Eca109 and EC1 cells were treated with 1, 2, and 4 μM of celastrol or DMSO for 24 h. And then the cells were stained with Annexin-V-FITC and PI apoptosis kit and tested by the flow cytometric analysis. Data were assessed with GraphPad Prism 8 software. **(C)** Celastrol increased the expression level of c-PARP. Eca109 and EC1 cells were treated with 1, 2, and 4 μM celastrol or DMSO for 24 h, and cell lysates were analyzed by Western blotting with a specific antibody against c-PARP with β-actin as a loading control. **(D)** ATPlite assay was used to determine the cell viability of Eca109 and EC1 cells treated with DMSO, 4 μM celastrol, 20 μM Z-VAD-FMK, or both for 24 h. **(E)** Eca109 and EC1 cells treated with DMSO, 4 μM celastrol, 20 μM Z-VAD-FMK, or both for 24 h, and cell lysates were analyzed by Western blotting using an antibody against c-PARP with β-actin as a loading control. ^∗^denotes *p* < 0.05, ^∗^^∗^^∗^denotes *p* < 0.001, n.s. denotes not significant.

In order to explore the mechanism of celastrol inhibiting the viability of ESCC cells, we determined the cellular response elicited by celastrol. We observed an obvious feature of apoptosis-shrunk morphology of ESCC cells under the treatment of celastrol (data not shown). Annexin V-FITC/PI double-staining analysis was further used to verify whether celastrol induced apoptosis in ESCC cells. As shown in Figures 2A,B, celastrol treatment resulted in a remarkable increase in the apoptotic cell population. Furthermore, we detected the expression of c-PARP, the classical marker of apoptosis. As shown, the expression level of c-PARP was obviously upregulated upon celastrol stimulation (Figure 2C). In addition, we found that apoptosis inhibitor Z-VAD-FMK alleviated the inhibition of celastrol on the viability of ESCC cells (Figures 2D,E). These results collectively demonstrated that celastrol inhibited the growth of ESCC cells by triggering apoptosis.

### Celastrol Induced DR5-Dependent Extrinsic Apoptosis by Transcriptional Activation of ATF4

**FIGURE 3:**
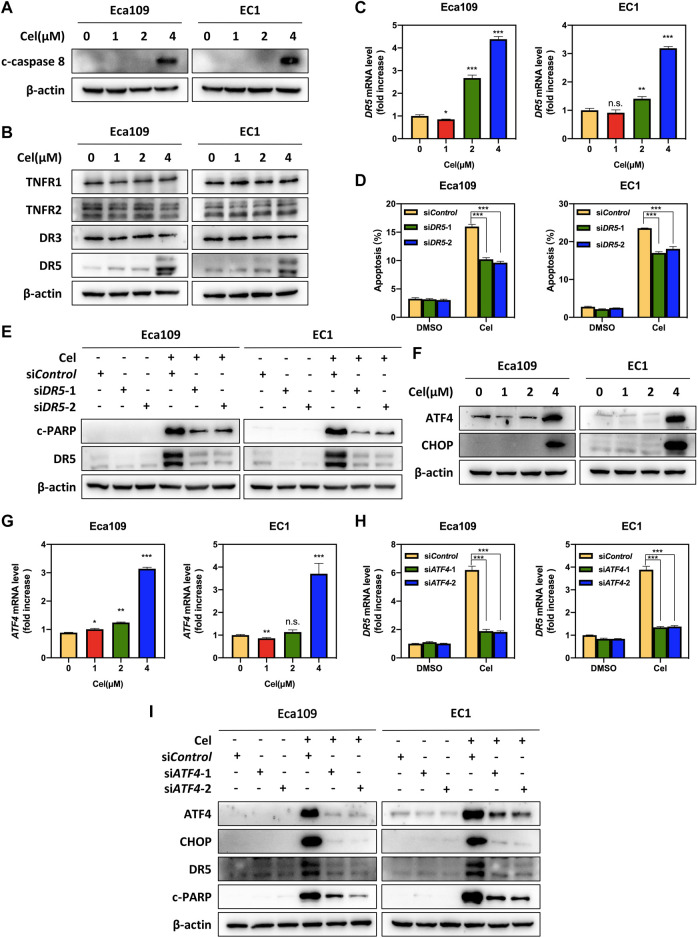
Celastrol activated DR5-dependent extrinsic apoptosis by upregulating ATF4. **(A)** Celastrol induced the activation of caspase 8. Eca109 and EC1 cells were treated with 1, 2, and 4 μM of celastrol or DMSO for 24 h, and cell lysates were analyzed by Western blotting using a specific antibody against c-caspase 8 with β-actin as a loading control. **(B)** Celastrol activated extrinsic apoptosis by upregulated DR5. Eca109 and EC1 cells were treated with 1, 2, and 4 μM of celastrol or DMSO for 24 h, and cell lysates were analyzed by Western blotting using specific antibodies against TNFR1, TNFR2, DR3, and DR5 with β-actin as a loading control. **(C)** Celastrol increased the mRNA level of *DR5*. Eca109 and EC1 cells were treated with 1, 2, and 4 μM of celastrol or DMSO for 24 h, and the mRNA level of *DR5* was determined by the real-time PCR. **(D,E)** Knockdown of DR5 inhibited apoptosis induced by celastrol. Eca109 and EC1 cells were transfected with control or si*DR5* for 72 h, and then treated with 4 μM celastrol or DMSO for 24 h. Apoptosis induction was quantified by Annexin V-FITC/PI double-staining analysis. Cell lysates were analyzed by Western blotting using specific antibodies against c-PARP and DR5 with β-actin as a loading control. **(F)** Celastrol induced the accumulation of ATF4 and CHOP. Eca109 and EC1 cells were treated with 1, 2, and 4 μM of celastrol or DMSO for 24 h. Cell lysates were analyzed by Western blotting using antibodies against ATF4 and CHOP with β-actin as a loading control. **(G)** Celastrol increased the mRNA level of *ATF4*. Eca109 and EC1 cells were treated with1 μM, 2, and 4 μM of celastrol or DMSO for 24 h, and the mRNA level of *ATF4* was determined by the real-time PCR. **(H,I)** Celastrol induced apoptosis of Eca109 and EC1 cells via the ATF4-DR5 axis. Eca109 and EC1 cells were transfected with control or si*ATF4* for 72 h, and then treated with 4 μM celastrol or DMSO for 24 h. The effect of si*ATF4* on *DR5* transcription was analyzed by real-time PCR. Expression levels of ATF4, CHOP, DR5, and c-PARP were assessed by Western blotting with β-actin as a loading control. ^∗^denotes *p* < 0.05, ^∗^^∗^denotes *p* < 0.01, ^∗^^∗^^∗^denotes *p* < 0.001, n.s. denotes not significant.

To further characterize the mechanism underlying celastrol-induced apoptosis, we determined the expression of c-caspase 8, a marker of extrinsic apoptosis. Our data showed that celastrol upregulated the expression of c-caspase 8 in Eca109 and EC1 cells, indicating that celastrol activated extrinsic apoptosis (Figure 3A). In order to explore the activation mechanism of extrinsic (death receptor-mediated) apoptosis under celastrol treatment, we evaluated the expression of death receptor family members. Among these death receptors (TNRF1, TNRF2, DR3, and DR5), the protein and mRNA levels of DR5 were significantly increased elicited by celastrol (Figures 3B,C). To further define the role of DR5 in celastrol-induced apoptosis, the expression of *DR5* was downregulated using two siRNA sequences. As shown, DR5 knockdown significantly attenuated the percentage of apoptotic cells in ESCC cells (Figure 3D and Supplementary Figure S1A), and downregulated the expression of c-PARP (Figure 3E). These results suggested that celastrol-triggered extrinsic apoptosis was mediated by DR5.

Previous studies reported that anti-cancer agents (e.g., MLN4924) transcriptionally activated ATF4 by inducing ER stress, and subsequently induced CHOP-mediated *DR5* transcription and caspase 8-mediated extrinsic apoptosis (Chen et al., 2016). Therefore, we speculated celastrol activated extrinsic apoptosis through ATF4-DR5 axis. To verify this hypothesis, we first determined the expression of ATF4 and CHOP, and the results showed that celastrol significantly upregulated the expression of ATF4 and CHOP at both protein and mRNA levels (Figures 3F,G and Supplementary Figure S1B). Next, we determined whether DR5-induced extrinsic apoptosis elicited by celastrol was dependent on ATF4. Our results showed that ATF4 knockdown significantly decreased the mRNA and protein levels of CHOP and DR5, indicating that ATF4 transactivated CHOP and DR5 upon celastrol stimulation (Figures 3H,I and Supplementary Figure S1C). Meanwhile, the knockdown of ATF4 obviously downregulated the expression of c-PARP (Figure 3I). Taken together, these findings demonstrated that celastrol activated extrinsic apoptosis of ESCC cells through the ATF4-DR5 axis.

### ATF4 Meditated Celastrol-Induced Noxa Upregulation

**FIGURE 4:**
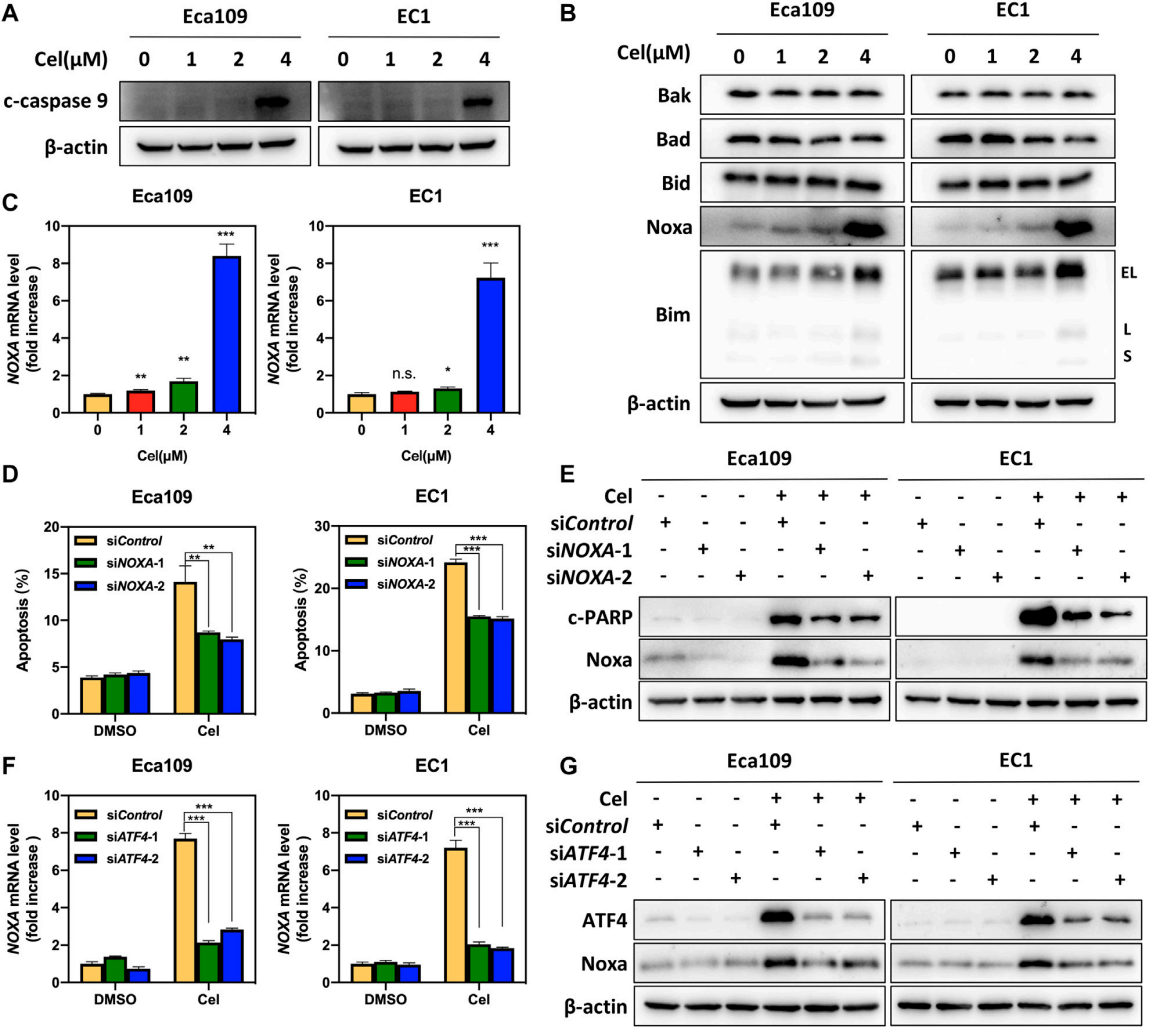
ATF4 meditated celastrol-induced Noxa upregulation. **(A)** Celastrol induced the activation of caspase 9. Eca109 and EC1 cells were treated with 1, 2, and 4 μM of celastrol or DMSO for 24 h, and cell lysates were analyzed by Western blotting using a specific antibody against c-caspase 9 with β-actin as a loading control. **(B)** Celastrol activated extrinsic apoptosis by upregulating Noxa and Bim. Eca109 and EC1 cells were treated with 1, 2, and 4 μM of celastrol or DMSO for 24 h, and then cell lysates were analyzed by Western blotting with specific antibodies against Bak, Bad, Bid, Noxa, and Bim with β-actin as a loading control. Three major Bim isoforms were created by alternative splicing: BimS, BimL, and BimEL. **(C)** Celastrol increased the mRNA level of *NOXA*. Eca109 and EC1 cells were treated with 1, 2, and 4 μM of celastrol or DMSO for 24 h, and the mRNA level of *NOXA* was determined by real-time PCR. **(D)** Knockdown of Noxa inhibited apoptosis induced by celastrol. Eca109 and EC1 cells were transfected with control or si*NOXA* for 72 h, and then treated with 4 μM celastrol or DMSO for 24 h. Apoptosis induction was quantified by Annexin V-FITC/PI double-staining analysis. **(E)** Cell lysates were analyzed by Western blotting using specific antibodies against c-PARP and Noxa with β-actin as a loading control. **(F,G)** ATF4 meditated celastrol-induced Noxa upregulation. Eca109 and EC1 cells were transfected with control or si*ATF4* for 72 h, and then treated with 4 μM celastrol or DMSO for 24 h. The effect of si*ATF4* on *NOXA* transcription was analyzed by real-time PCR. Expression levels of ATF4 and Noxa were assessed by Western blotting analysis with β-actin as a loading control. ^∗^denotes *p* < 0.05, ^∗^^∗^denotes *p* < 0.01, ^∗^^∗^^∗^denotes *p* < 0.001, n.s. denotes not significant.

To investigate whether celastrol induced intrinsic apoptosis, we determined the expression of c-caspase 9 in ESCC cells exposed to celastrol. As shown, the expression level of c-caspase 9 remarkably upregulated in both two ESCC cell lines (Figure 4A). In order to illustrate the mechanism underlying celastrol-induced intrinsic apoptosis, we examined the expression of classical pro-apoptotic proteins, including Bax, Bak, Bid, Noxa, and Bim. As shown, after celastrol treatment, we observed the expression levels of Noxa and Bim, two pro-apoptotic BH3-only members (Kale et al., 2017), strikingly elevated in Eca109 and EC1 cells, while the expression of Bax, Bak, and Bid did not change (Figures 4B,C), suggesting that celastrol activated Noxa and Bim. To further define the role of Noxa in celastrol-induced intrinsic apoptosis, the expression of *NOXA* was downregulated by siRNA silencing in celastrol-treated cells. As shown, Noxa knockdown significantly reduced the induction of apoptosis and the cleavage of PARP, highlighting a critical role of Noxa in celastrol-induced intrinsic apoptosis (Figures 4D,E and Supplementary Figure S1D). Given that Noxa was known to be regulated by ATF4, we, therefore, tested the involvement of ATF4 in celastrol-induced Noxa expression (Sharma et al., 2018). Indeed, downregulation of ATF4 significantly suppressed the induction of Noxa at both mRNA and protein levels (Figures 4F,G), supporting the notion that ATF4 meditated celastrol-induced Noxa upregulation.

### FoxO3a Meditated Celastrol-Induced Bim Upregulation

**FIGURE 5:**
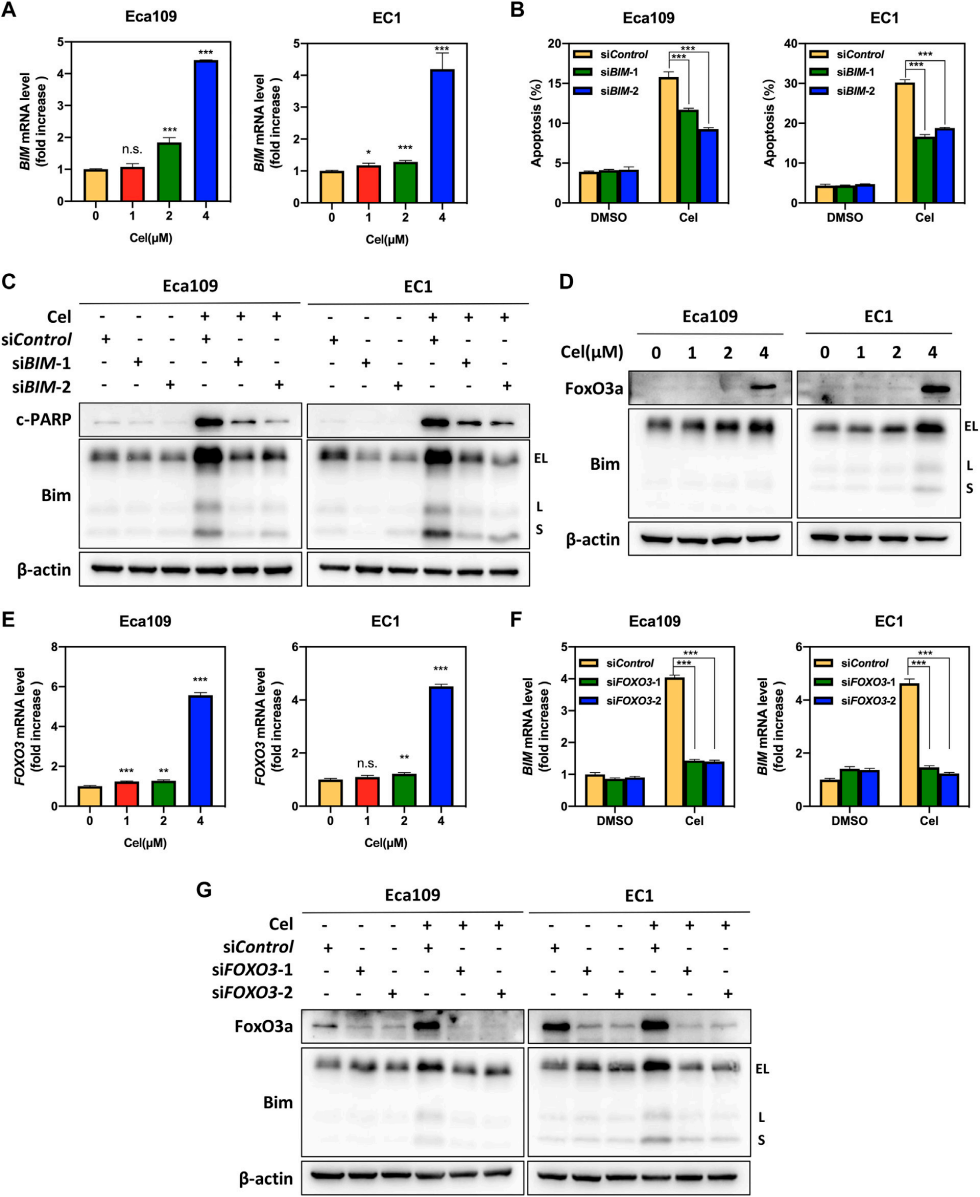
FoxO3a meditated celastrol-induced Bim upregulation. **(A)** Celastrol increased the mRNA level of *BIM*. Eca109 and EC1 cells were treated with 1, 2, and 4 μM of celastrol or DMSO for 24 h. The mRNA level of *BIM* was determined by real-time PCR. **(B,C)** Knockdown of Bim inhibited apoptosis induced by celastrol. Eca109 and EC1 cells were transfected with control or si*BIM* for 72 h, and then treated with 4 μM celastrol or DMSO for 24 h. Apoptosis induction was quantified by Annexin V-FITC/PI double-staining analysis. Cell lysates were analyzed by Western blotting using specific antibodies against c-PARP and Bim with β-actin as a loading control. Three major Bim isoforms were created by alternative splicing: BimS, BimL, and BimEL. **(D,E)** Celastrol induced the upregulation of FoxO3a. Eca109 and EC1 cells were treated with 1, 2, and 4 μM of celastrol or DMSO for 24 h, and cell lysates were analyzed by Western blotting with an antibody against Bim and FoxO3a with β-actin as a loading control. Three major Bim isoforms were created by alternative splicing: BimS, BimL, and BimEL. The mRNA level of *FOXO3* was determined by real-time PCR. **(F,G)** FoxO3a was the response to celastrol-induced Bim upregulation. Eca109 and EC1 cells were transfected (72 h) with control or si*FOXO3*, and treated with 4 μM celastrol for 24 h. The effect of si*FOXO3* on *BIM* transcription was analyzed by real-time PCR. Expression levels of FoxO3a and Bim were assessed by Western blotting analysis with β-actin as a loading control. Three major Bim isoforms were created by alternative splicing: BimS, BimL, and BimEL. ^∗^denotes *p* < 0.05, ^∗∗^denotes *p* < 0.01, ^∗∗∗^denotes *p* < 0.001, n.s. denotes not significant.

Our aforementioned results indicated that celastrol upregulated the expression of the pro-apoptotic protein Bim (Figure 4B). We further found that the mRNA level of *BIM* was significantly elevated upon celastrol treatment (Figure 5A). To further determine the potential role of Bim in celastrol-mediated intrinsic apoptosis, the expression of *BIM* was downregulated via siRNA silencing. Our data showed that knockdown of Bim significantly alleviated the percentage of apoptotic cells induced by celastrol (Figure 5B and Supplementary Figure S1E), along with a reduction of the c-PARP (Figure 5C), demonstrating that Bim was involved in celastrol-induced intrinsic apoptosis of ESCC cells. Considering that *FOXO3*, one of forkhead transcription factor family, is the notable transcription factor regulating *BIM* gene expression in response to apoptosis (Zhenxing et al., 2019). We, therefore, examined the potential role of FoxO3a in celastrol-induced Bim expression. As shown in Figures 5D,E, celastrol treatment significantly upregulated the mRNA and protein levels of FoxO3a in ESCC cells. Furthermore, we found that downregulation of FoxO3a significantly inhibited the accumulation of Bim induced by celastrol (Figures 5F,G), illustrating that FoxO3a meditated celastrol-induced Bim upregulation. Collectively, our findings demonstrated that celastrol activated intrinsic apoptosis of ESCC cells via the ATF4-Noxa and FoxO3a-Bim axis.

### Celastrol Suppressed the Growth of Esophageal Squamous Cell Carcinoma *in vivo*

**FIGURE 6:**
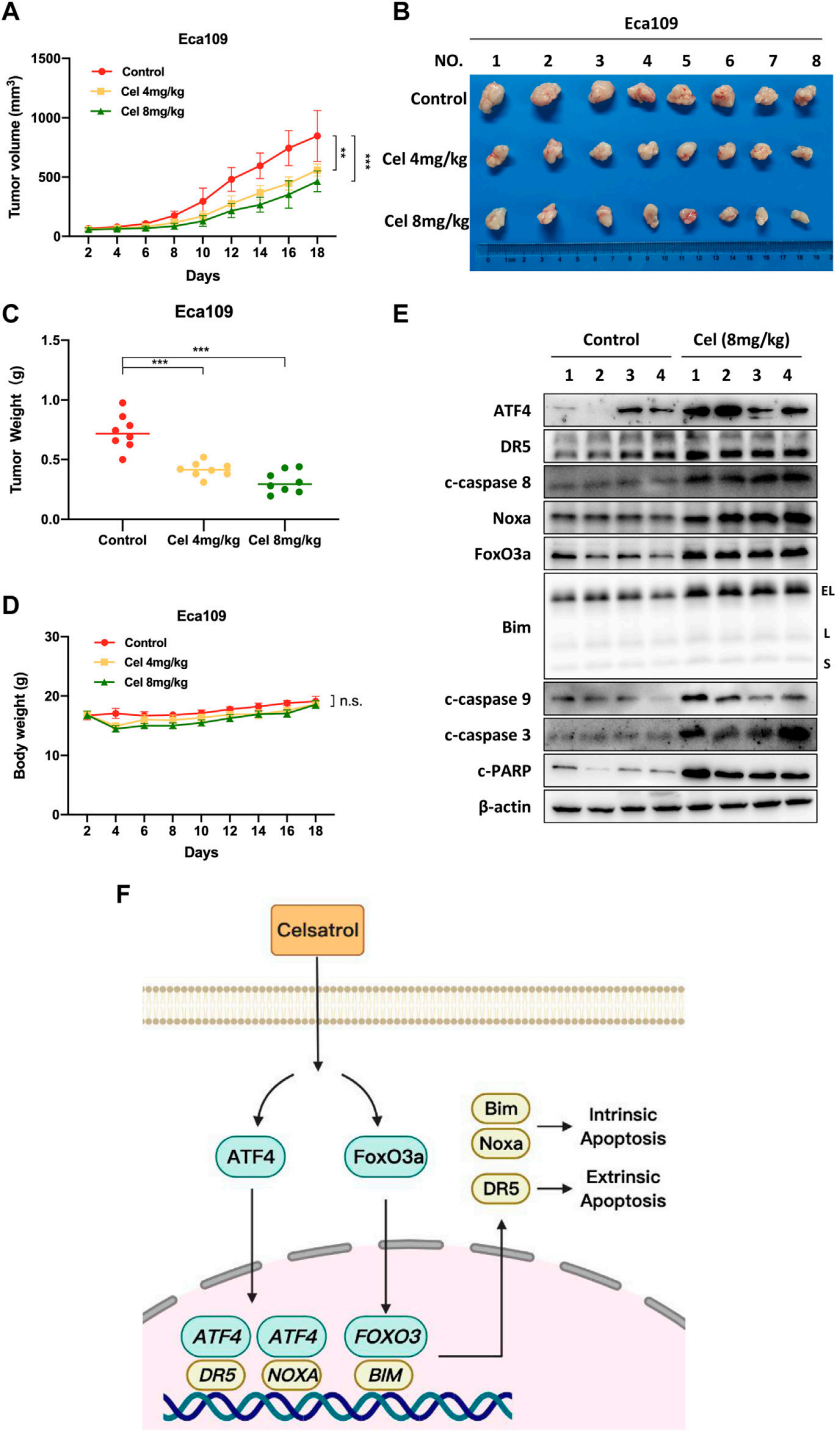
Celastrol suppressed the growth of ESCC *in vivo*. **(A)** Nude mice have subcutaneously transplanted Eca109 cells and are treated with celastrol as described in Materials and Methods. Tumor size was determined with a caliper every other day, and the volume was calculated to construct a growth curve. **(B)** Mice were sacrificed, and tumor tissues were harvested and photographed. **(C)** The tumor weight was measured with an electronic scale on the sacrificed day. **(D)** Body weight of each mouse was recorded every other day during the whole experiment. **(E)** Celastrol induced extrinsic and intrinsic apoptosis *in vivo*. Proteins extracted from tumor tissues were analyzed by Western blotting using specific antibodies against ATF4, DR5, c-caspase 8, Noxa, FoxO3a, Bim, c-caspase 9, c-caspase 3, and c-PARP with β-actin as a loading control. Three major Bim isoforms were created by alternative splicing: BimS, BimL, and BimEL. **(F)** The mechanism of celastrol inhibits the tumor growth of ESCC. Celastrol coordinatively triggered DR5-dependent extrinsic apoptosis and Noxa-dependent intrinsic apoptosis through transcriptional activation of ATF4. FoxO3a-Bim pathway contributed to celastrol-induced intrinsic apoptosis in ESCC cells. ^∗∗^denotes *p* < 0.01, ^∗∗∗^denotes *p* < 0.001, n.s. denotes not significant.

Finally, we established a subcutaneous transplantation tumor model with Eca109 cells to examine the anti-tumor potential of celastrol *in vivo*. Compared with the control group, celastrol significantly inhibited the tumor growth over time (4 and 8 mg/kg celastrol corresponded to *p* < 0.01 and *p* < 0.001 respectively, Figure 6A). Moreover, the tumor weights of the celastrol-treated mice were much lower than those of the control mice (*p* < 0.001, Figures 6B,C). During the whole experiment, there was no significant change in animal weights (Figure 6D) and no significant morphological difference in liver and kidney (data not shown) between the celastrol-treated group and the control group. In addition, as shown in Figure 6E, celastrol triggered extrinsic and intrinsic apoptosis *in vivo*, as evidenced by the accumulation of apoptosis-related proteins in celastrol-treated tumor tissue, including ATF4, DR5, c-caspase 8, Noxa, FoxO3a, Bim, c-caspase 9, as well as c-caspase 3 and c-PARP. Together, our findings indicated that celastrol activated extrinsic and intrinsic apoptosis, thus inhibiting the tumor growth of ESCC both *in vitro* and *in vivo*.

## Discussion

ESCC is a highly malignant tumor of the digestive system, and its incidence and mortality rates are rising rapidly (Sung et al., 2021). In recent years, although some progress has been made in the diagnosis and treatment of ESCC, effective therapeutic strategies are still insufficient (Yang et al., 2020). An increasing body of evidence suggested that the natural products exhibited the potential anti-tumor efficacy in ESCC (Ying et al., 2018). In our study, we validated that celastrol was a promising candidate for the treatment of ESCC. We showed that celastrol significantly suppressed the malignant proliferation of ESCC cells, and strikingly inhibited the tumor growth in nude mouse xenograft model. Mechanistically, we demonstrated that celastrol coordinatively triggered DR5-dependent extrinsic apoptosis and Noxa-dependent intrinsic apoptosis through transcriptional activation of ATF4. Furthermore, we revealed that the FoxO3a-Bim pathway contributed to celastrol-induced intrinsic apoptosis of ESCC cells (Figure 6F). Our findings demonstrated the substantial inhibitory effect of celastrol on ESCC, which provided an attractive choice for the ESCC treatment.

It was reported that ER stress upregulated the expression of ATF4 and CHOP in response to unfolded protein response (UPR) (Kim et al., 2021). Death receptor DR5, a downstream target of CHOP, was activated after CHOP accumulation, and further triggered apoptosis cascades (Chen et al., 2016). In our study, we found that celastrol activated extrinsic apoptosis through the ATF4-CHOP-DR5 pathway, as evidenced by significantly diminished the expression of CHOP and DR5 after ATF4 knockdown. Except for CHOP and DR5, we found that ATF4 knockdown reduced celastrol-induced Noxa accumulation as well, suggesting that celastrol triggered Noxa-dependent intrinsic apoptosis by transcriptionally activating ATF4. Studies have shown that natural products such as parthenolide and curcumin activate eIF2α through ER stress, which in turn activates ATF4 and triggers apoptosis (Wu et al., 2010; Zhao et al., 2014). Therefore, celastrol might also activate ATF4 through ER stress. The mechanism by which celastrol transcriptionally activated ATF4 needs to be further clarified. Furthermore, our data showed that celastrol up-regulated the expression of p53, which was known to transcriptionally regulate Noxa expression as well (Supplementary Figure S2B) (Furukawa et al., 2018). Therefore, apart from ATF4, p53 may also be involved in the upregulation of Noxa induced by celastrol. Interestingly, in addition to Noxa, we found Bim contributed to celastrol-induced intrinsic apoptosis of ESCC cells, and FoxO3a meditated celastrol-induced Bim upregulation. However, FoxO3a knockdown did not completely alleviate celastrol-induced Bim accumulation. Given that other transcription factors (*Smad3*, *E2F1*, *JNK*/*c-Jun,* and *c-Myc*, etc) that are known to mediate Bim expression, whether these transcription factors are involved in the Bim induction elicited by celastrol remains further exploration (Chen et al., 2014; Muthalagu et al., 2014; Shats et al., 2017; Wildey et al., 2021).

The rapid growth of cancer cells is attributed to the accelerated cell cycle process (Zheng et al., 2019). In view of this, the blockage of the cell cycle process is considered an effective strategy to halt tumor growth (Thu et al., 2018). Previous studies have reported that celastrol prevented tumor cell proliferation by inducing cell cycle arrest (Ni et al., 2019). We found that celastrol treatment increased the cell populations in the G2/M phase of the cell cycle in ESCC cells (Supplementary Figure S2A). Furthermore, we showed that celastrol significantly upregulated the expression of the mitotic marker p-histone 3 (p-H3, ser10) and downregulated the expression of G2 phase marker p-cdc2, suggesting that celastrol induced ESCC cells to pass through G2/M checkpoint and then arrest at M phase. In addition, we found that celastrol caused the accumulation of cell cycle inhibitory protein p21 and its upstream protein p53, indicating that celastrol induced M-phase cell cycle arrest through the p53/p21 signaling pathway (Supplementary Figure S2B). It is well known that the best-characterized p53 function is the response to acute DNA damage (Boutelle and Attardi, 2021). Our data showed that celastrol significantly upregulated the expression of DNA damage marker p-H2AX, suggesting that celastrol initiated DNA damage and the activation of the p53/p21 signaling pathway, and then promoted cell cycle arrest (Supplementary Figure S2B). In fact, the persistence of DNA damage will induce programmed cell death such as apoptosis or cell senescence (Roos et al., 2015; Ou and Schumacher, 2018). Therefore, in our study, it is possible that celastrol caused the persistence of cell DNA damage during cell cycle arrest, which eventually led to apoptosis of ESCC cells. Furthermore, our results showed that caspase inhibitor Z-VAD-FMK only partially rescued the cellular viability after celastrol treatment, indicating that celastrol inhibited the proliferation of ESCC cells by inducing the combinatory effects of both cell cycle arrest and apoptosis.

In summary, our study revealed a previously unknown inhibitory efficacy of celastrol on ESCC by activating DR5-dependent extrinsic and Noxa/Bim-dependent intrinsic apoptosis, suggesting that celastrol was a candidate for apoptosis inducer in recalcitrant human ESCC.

## Data Availability

The original contributions presented in the study are included in the article/Supplementary Material, further inquiries can be directed to the corresponding author.
