# Supplementary material for: Celastrol Inhibited Human Esophageal Cancer by Activating DR5-Dependent Extrinsic and Noxa/Bim-Dependent Intrinsic Apoptosis
Source: Front Pharmacol. 2022 Jun 8;13:873166. doi: 10.3389/fphar.2022.873166 (PMC9219015; doi:10.3389/fphar.2022.873166)
Supplement: Supplementary file 1 [file DataSheet1.pdf]

·*Supplementary Material*

1 Supplementary Figures

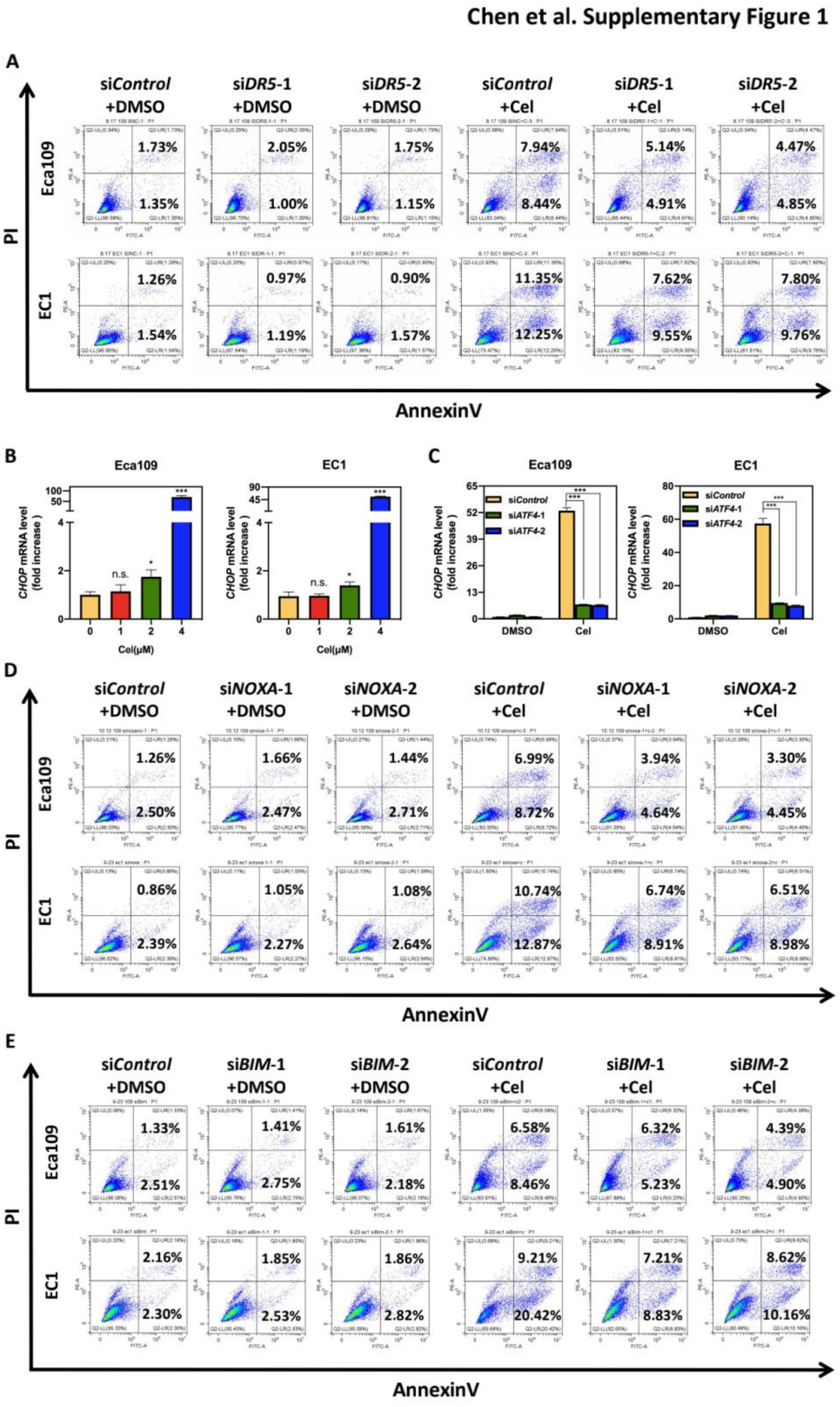

Supplementary Figure 1. (A) Knockdown of DR5 inhibited apoptosis induced by celastrol. Eca109 and EC1 cells were transfected with control or si*DR5* RNA (72 hours), and then treated with 4 μM celastrol or DMSO for 24 hours. Apoptosis induction was quantified by Annexin V-FITC/PI double-staining analysis. **(B)** Celastrol increased the mRNA level of *CHOP*. Eca109 and EC1 cells were treated with 1 μM, 2 μM and 4 μM of celastrol or DMSO for 24 hours, and the mRNA level of *CHOP* was determined by real-time PCR. **(C)** ATF4 meditated the transcription of CHOP. Eca109 and EC1 cells were transfected (72 hours) with control or si*ATF4*, treated with 4 μM celastrol or DMSO for 24 hours. The effect of si*ATF4* on CHOP transcription was analyzed by real-time PCR. **(D)** Knockdown of Noxa inhibited apoptosis induced by celastrol. Eca109 and EC1 cells were transfected with control or si*NOXA* (72 hours), and then treated with 4 μM celastrol or DMSO for 24 hours. Apoptosis induction was quantified by Annexin V-FITC/PI double-staining analysis. **(E)** Knockdown of Bim inhibited apoptosis induced by celastrol. Eca109 and EC1 cells were transfected with control or si*BIM* (72 hours), and then treated with 4 μM celastrol or DMSO for 24 hours. Apoptosis induction was quantified by Annexin V-FITC/PI double-staining analysis. *denotes $P < 0.05$, ***denotes $P < 0.001$, n.s. denotes not significant.

Chen et al. Supplementary Figure 2

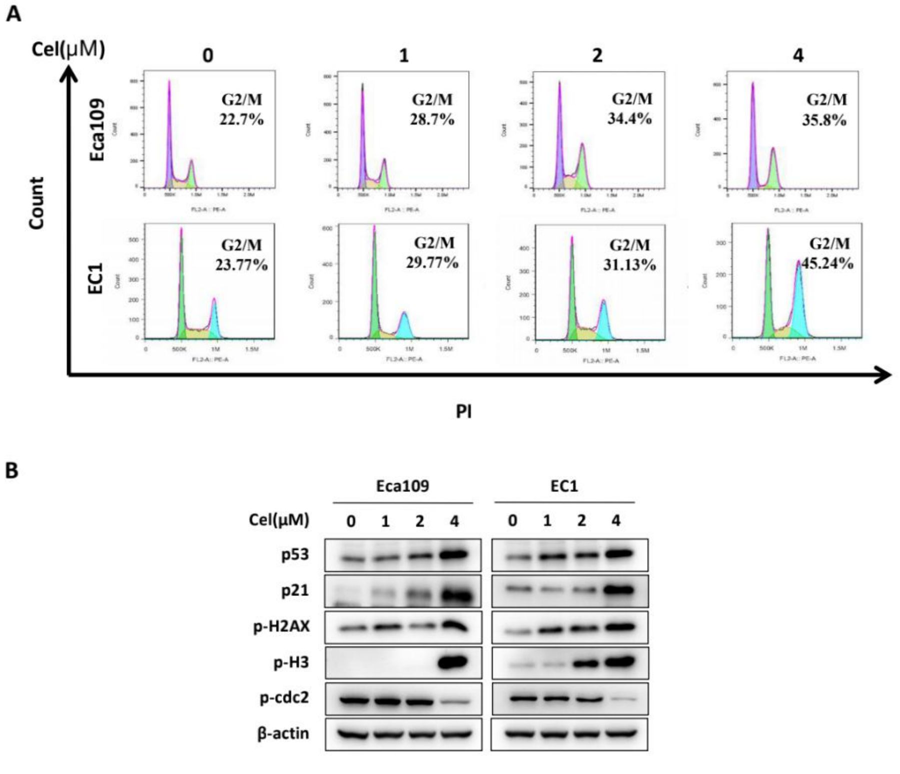

Supplementary Figure 2. (A) Celastrol arrested cell cycle at M-phase in ESCC cells. Eca109 and EC1 cells were pre-incubated with 1 μM, 2 μM and 4 μM of celastrol or DMSO for 24 hours, and then the cells were incubated by PI, detected by the flow cytometric analysis. Cell cycle was analyzed with FlowJo 8 software. **(B)** Celastrol induced the accumulation of p53, p21, p-H2AX, p-H3, and a decrease in p-cdc2. Eca109 and EC1 cells were treated with 1 μM, 2 μM and 4 μM of celastrol or DMSO for 24 hours. Cell lysates were assessed by Western blotting using antibodies against p53, p21, p-H2AX, p-H3 and p-cdc2 with β-actin as a loading control.
